# Catalytic Knockdown of miR-21 by Artificial Ribonuclease: Biological Performance in Tumor Model

**DOI:** 10.3389/fphar.2019.00879

**Published:** 2019-08-08

**Authors:** Olga A. Patutina, Svetlana K. Miroshnichenko, Nadezhda L. Mironova, Aleksandra V. Sen’kova, Elena V. Bichenkova, David J. Clarke, Valentin V. Vlassov, Marina A. Zenkova

**Affiliations:** ^1^Laboratory of Nucleic Acids Biochemistry, Institute of Chemical Biology and Fundamental Medicine SB RAS, Novosibirsk, Russia; ^2^School of Health Sciences, Faculty of Biology, Medicine and Health, University of Manchester, Manchester, United Kingdom

**Keywords:** artificial ribonuclease, oligonucleotide-peptide conjugate, RNA cleavage, RNase H, anti-miRNA therapy, miRNA-21

## Abstract

Control of the expression of oncogenic small non-coding RNAs, notably microRNAs (miRNAs), is an attractive therapeutic approach. We report a design platform for catalytic knockdown of miRNA targets with artificial, sequence-specific ribonucleases. miRNases comprise a peptide [(LeuArg)_2_Gly]_2_ capable of RNA cleavage conjugated to the miRNA-targeted oligodeoxyribonucleotide, which becomes nuclease-resistant within the conjugate design, without resort to chemically modified nucleotides. Our data presented here showed for the first time a truly catalytic character of our miR-21-miRNase and its ability to cleave miR-21 in a multiple catalytic turnover mode. We demonstrate that miRNase targeted to miR-21 (miR-21-miRNase) knocked down malignant behavior of tumor cells, including induction of apoptosis, inhibition of cell invasiveness, and retardation of tumor growth, which persisted on transplantation into mice of tumor cells treated once with miR-21-miRNase. Crucially, we discover that the high biological activity of miR-21-miRNase can be directly related not only to its truly catalytic sequence-specific cleavage of miRNA but also to its ability to recruit the non-sequence specific RNase H found in most cells to elevate catalytic turnover further. miR-21-miRNase worked synergistically even with low levels of RNase H. Estimated degradation in the presence of RNase H exceeded 10^3^ miRNA target molecules per hour for each miR-21-miRNase molecule, which provides the potency to minimize delivery requirements to a few molecules per cell. In contrast to the comparatively high doses required for the simple steric block of antisense oligonucleotides, truly catalytic inactivation of miRNA offers more effective, irreversible, and persistent suppression of many copy target sequences. miRNase design can be readily adapted to target other pathogenic microRNAs overexpressed in many disease states.

## Introduction

The well-recognized function of miRNAs as effective biological regulators and their role in the development of a broad spectrum of human pathophysiology offer oncology both novel biomarkers (Markou et al., 2013; Tahiri et al., 2014) and a promising paradigm shift in therapeutic targets (Frixa et al., 2015; O’Bryan et al., 2017; Zhou et al., 2017) from the protein-coding genome or already-expressed pathogenic proteins (Lennox and Behlke, 2011; Gambari et al., 2016).

To date, several technologies have been proposed to suppress excessive miRNA expression and function. Deep analysis of the structure of the key proteins involved in synthesis and maturation of miRNA, along with a large-scale ligand screening, has allowed selection of small molecule inhibitors suppressing enzymes involved in miRNA biogenesis broadly (Gumireddy et al., 2008; Lorenz et al., 2015; Di Giorgio et al., 2016). Much more selectively, miRNA-masking oligonucleotides can prevent the interaction of a miRNA with its target mRNA (Choi et al., 2007; Xiao et al., 2007; Wang, 2011), and anti-miRNA oligonucleotides can knock down target miRNA through formation of complementary complexes (Lennox and Behlke, 2011; Lennox et al., 2017; Miroshnichenko et al., 2019). Given the high doses often necessary, interest has grown in mopping up miRNA with larger structures with multiple miRNA binding sites, such as miRNA sponges (Ebert et al., 2007; Jung et al., 2015) and miRNA zippers, which tandemly bind the 3′- and 5′-ends of two miRNAs, thus forming an extended duplex with multiple copies of bound miRNA molecules (Meng et al., 2017).

Genome editing with the CRISPR/Cas9 system to decrease the level of mature miRNA (Chang et al., 2016; Huo et al., 2017) might be a potential alternative to binding many miRNA copies present and replenished in target cells. However, only sequence-selective artificial ribonucleases (aRNases) offer the potential to degrade many copies of miRNA in the cell. RNA-cleavage agents conjugated to oligonucleotide recognition motifs can selectively recognize specific miRNA sequences and hydrolyze the phosphodiester bonds within RNA molecules. However, in order to act as true enzymes, such artificial ribonucleases must exhibit catalytic turnover, which requires rapid release of RNA fragments after each cleavage event, followed by many subsequent attacks of other target RNA molecules. Metal-ion dependent (Dy^3+^, Eu^3+^, Cu^2+^, Zn^2+^) ribonucleases showed some promising results in terms of sequence-specific cleavage of RNA in a catalytic regime (Magda et al., 1997; Häner et al., 1998; Trawick et al., 2001; Niittymäki et al., 2004; Niittymäki and Lönnberg, 2006; Murtola et al., 2010). However, these aRNases were not amenable to application *in vivo* because of competing protein ligands and other bioavailable metals in cells and tissue, which render them virtually uncontrollable. Moreover, metal loss from their coordinating ligands led to concerns about their toxicity in humans. Metal-independent artificial ribonucleases offer advantages including reduced toxicities and the ability to work in intracellular conditions. However, most of the developed metal-independent aRNases are less active and show low to negligible catalytic turnover (Mironova et al., 2006; Mironova et al., 2007). Hybrid enzymes engineered by the fusion of oligodeoxyribonucleotide to staphylococcal nuclease efficiently hydrolyzed complementary RNA targets but did not leave the substrate after cleavage (Zuckermann and Schultz, 1989). This was overcome by creating a hybrid of *Escherichia coli* RNase H and a 9-mer oligodeoxynucleotide, when the catalytic turnover of the reaction of sequence-specific cleavage was achieved (Kanaya et al., 1992), but never investigated *in vivo*.

Recently we have reported the discovery of novel miRNA-specific metal-independent aRNases, here called miRNases, capable of selective cleavage of biologically significant miRNAs (Patutina et al., 2017a), where we presented a design concept, synthesis, and full characterization of a panel of the bioconjugates at their discovery stage with the significant structural and functional variations. Each of those miRNases comprised an oligodeoxyribonucleotide complementary to the miRNA of interest as a recognition motif conjugated to peptide acetyl-[(LeuArg)_2_Gly]_2_ capable of a site-specific scission of phosphodiester bonds within the target miRNA molecule. That pilot study allowed us to compare *back-to-back* the ability of such conjugates to cleave the target miR-21 in a sequence-specific manner to distinguish successful structural candidates from inactive counterparts. One of the most efficient miRNases from that series (i.e. 5′-h-9/14) was used for preliminary evaluation of a biological activity in order to estimate the future antitumor potential of these catalytic bioconjugates. As the selected miRNase targeted to the oncogenic miR-21 (miR-21-miRNase) showed efficient sequence-selective cleavage, leading to inhibition of miR-21 in lymphosarcoma cells and suppression of tumor cell proliferation (Patutina et al., 2017a), we undertake here a comprehensive study of its serum stability and biological performance. We demonstrate that specific deactivation of miR-21 by miR-21-miRNase induces apoptosis in tumor cells, suppresses their invasive properties, and decreases the proliferative rate of tumor cells *in vitro*. We show here for the first time that a single treatment of tumor cells with miR-21-miRNase persisted to retard subsequent tumor growth *in vivo*. We report here the discovery that the high efficiency of miRNA inactivation in cells by this miRNase is related to its ability to work in a true catalytic mode with multiple turnover, which is increased considerably by recruitment of the non-sequence specific intracellular RNase H.

## Materials and Methods

### Peptide-Oligonucleotide Conjugates (POCs)

Oligodeoxyribonucleotides (with and without an aminohexyl linker attached to the 5′-terminal phosphate of the oligonucleotide, and 2′-O-methyl-modification (2′-OMe) (see **Supplementary Table 1**) were synthesized in ICBFM SB RAS (Russia) by Dr. V. Ryabinin and Dr. Maria Meschaninova. The catalytic peptide, acetyl-[(LeuArg)_2_Gly]_2_, was attached *via* its C-terminus to the amine group of the aminohexyl linker located at the 5′-terminus of respective oligonucleotides to prepare the miR-21-miRNase and luc-POC conjugates (Patutina et al., 2017a).

### 5′-RNA Labeling

miR-21, 5′-UAGCUUAUCAGACUGAUGUUGA-3′, was 5′-end labeled using γ-[^32^P]-ATP and T4 polynucleotide kinase (Thermo Scientific, USA) as previously described (Silberklang et al., 1979; Mironova et al., 2007).

### Assay of Ribonuclease Activity and miR-21-miRNase in Multiple Turnover Conditions

Unlabeled miR-21 (10 µM, 25 µM, or 50 µM) and 10^5^ cpm (Cherenkov’s counting) of [^32^P]-miR-21 was incubated at 37°C with either miR-21-miRNase (5 µM) or RNase H (100 U/ml, plus 5 µM of either h-ODN or miR-21-miRNase) in buffer (20 mM Tris-HCl, pH 7.8, 40 mM KCl, 8 mM MgCl_2_, 1 mM DTT). Reactions were quenched by precipitation of RNA with 2% LiClO_4_ in acetone, and RNA cleavage products quantified in dried gels as described previously (Patutina et al., 2017a) using Molecular Imager FX and Quantity One software.

### Nuclease Resistance Assay

Oligonucleotides or conjugates (0.1 µg/µl) were incubated at 37°C in Dulbecco’s Modified Eagle Medium (DMEM) (Sigma, USA) with 10% fetal bovine serum (FBS; GE Healthcare, USA). Reactions were quenched in urea (8M) and immediately frozen in liquid nitrogen, prior to analysis of thawed samples in 12% PAAG/8M urea gels, using Tris-Borate-EDTA (TBE) as running buffer, Stains-All (MP Biomedicals, USA) and photographic gel documentation (VilberLourmat, France).

### Cell Lines

Mouse B16 melanoma cells, grown in DMEM/10% FBS/1% antibiotic antimycotic solution (ICN, Germany) and RLS_40_ lymphosarcoma cells, grown in IMDM/10% FBS/1% antibiotic antimycotic solution, were cultivated at 37°C in a humidified incubator with 5% CO_2_.

### Transfection of Cells With Oligonucleotides and Conjugates

B16 cells were pre-seeded (2 × 10^5^ per well of 24-well plates) in DMEM/10% FBS a day before transfection. Medium was replaced with serum-free DMEM, prior to incubation for 4 h with h-ODN, 2′-OMe, Inh (hsa-miR-21 5 p inhibitor, Ambion, USA), miR-21-miRNase, or luc-POC, each (0.1 or 1 µM) pre-complexed with Lipofectamine (Thermo Fisher Scientific, USA) in Opti-MEM medium (Thermo Fisher Scientific, USA) according to manufacturer’s instructions, then cells were cultivated in fresh culture standard medium for 24–72 h.

### Annexin V-FITC/PI Apoptosis Assay

At 24, 48, and 72 h after transfection, cells were harvested, washed twice with PBS, resuspended in Binding Buffer to a density of 2–5 × 10^5^/ml, and incubated with annexin V-FITC and propidium iodide (PI) (ab14085, Abcam, UK) in the dark at room temperature for 15 min, prior to flow cytometry analysis (Novocyte CEA Biosciences, USA).

### *In Vitro* Invasiveness Assay

Real-time cell analysis (xCELLigence, ACEABiosciences, USA) used CIM-Plates held at 37°C under an atmosphere of 5% CO_2_. Top chamber wells were coated with Matrigel (20 µl per well, diluted 1:40 with cold serum-free DMEM) and allowed to polymerize (4 h at 37°C under 5% CO_2_) prior to cell seeding. Bottom chamber wells were filled with DMEM/10% FBS (160 µl each), prior to assembly of the chambers, when serum-free DMEM was added to the top wells (30 µl each) and equilibrated (1 h at 37°C under 5% CO_2_). At 4 h after transfection, B16 cells (4 × 10^4^) were seeded into each top chamber well in serum-free DMEM and pre-incubated for 30 min at room temperature before monitoring (xCELLigence set to collect impedance data, reported as cell index at least once every 30 min).

### Mice

Male 10–12 week-old CBA/LacSto (hereinafter, CBA) mice were kept in the vivarium of the Institute of Chemical Biology and Fundamental Medicine, SB RAS, with a natural light regime on a standard diet for laboratory animals [GOST (State Standard) R 5025892] in compliance with the international recommendations of the European Convention for the Protection of vertebrate animals used for experimental studies (1997), as well as the rules of laboratory practice in the performance of pre-clinical studies in the Russian State Standards (R 51000.3–96 and 51000.4–96). The experimental protocols were approved by the Committee on the Ethics of Animal Experiments with the Institute of Cytology and Genetics of the Siberian Branch of the Russian Academy of Sciences.

### Tumor Transplantation Assay

RLS_40_ ascites were taken from CBA mice intraperitoneally injected with tumor cells (2 × 10^6^ in 0.2 ml buffered saline) into their abdominal cavities. RLS_40_ cells, isolated from ascites fluid by filtration through LSM were divided into seven portions: 1) untreated cells; 2) the cells incubated with Lipofectamine ; the cells transfected with 1 µM of 3) h-ODN, 4) 2′-OMe, 5) Inh, 6) miR-21-miRNase, or 7) luc-POC each. After 4 h of transfection, cell suspensions (0.1 ml, 2 × 10^6^ cells) were intramuscularly inoculated into the right lower limb of CBA mice for solid tumor development. As soon as tumors began to be palpable, the tumor volumes were measured every 2–3 days using calipers. Tumor volumes were calculated as V = (π/6 × length × width × height). Hepatic index was estimated as (liver weight/mouse weight) × 100% and as 5.0% for healthy CBA mice. Tumor doubling time (DT) was estimated as DT = (*t* − *t*0) × ln 2/(ln *V* − ln *V*0), where (*t* − *t*0) indicates the length of time between two measurements of tumor size and *V*0 and *V* denote the tumor volume at two points of the measurement (Ozono et al., 2004).

## Statistics

Data were statistically processed using two-way ANOVA taking into account two factors: antisense oligonucleotide and peptide. *Post hoc* testing was completed using Tukey test; *p* < 0.05 was considered to be statistically significant. The statistics package STATISTICA version 12.0 was used for this analysis.

## Results

### Structure of Nuclease-Resistant miR-21-miRNase

**Figure 1:**
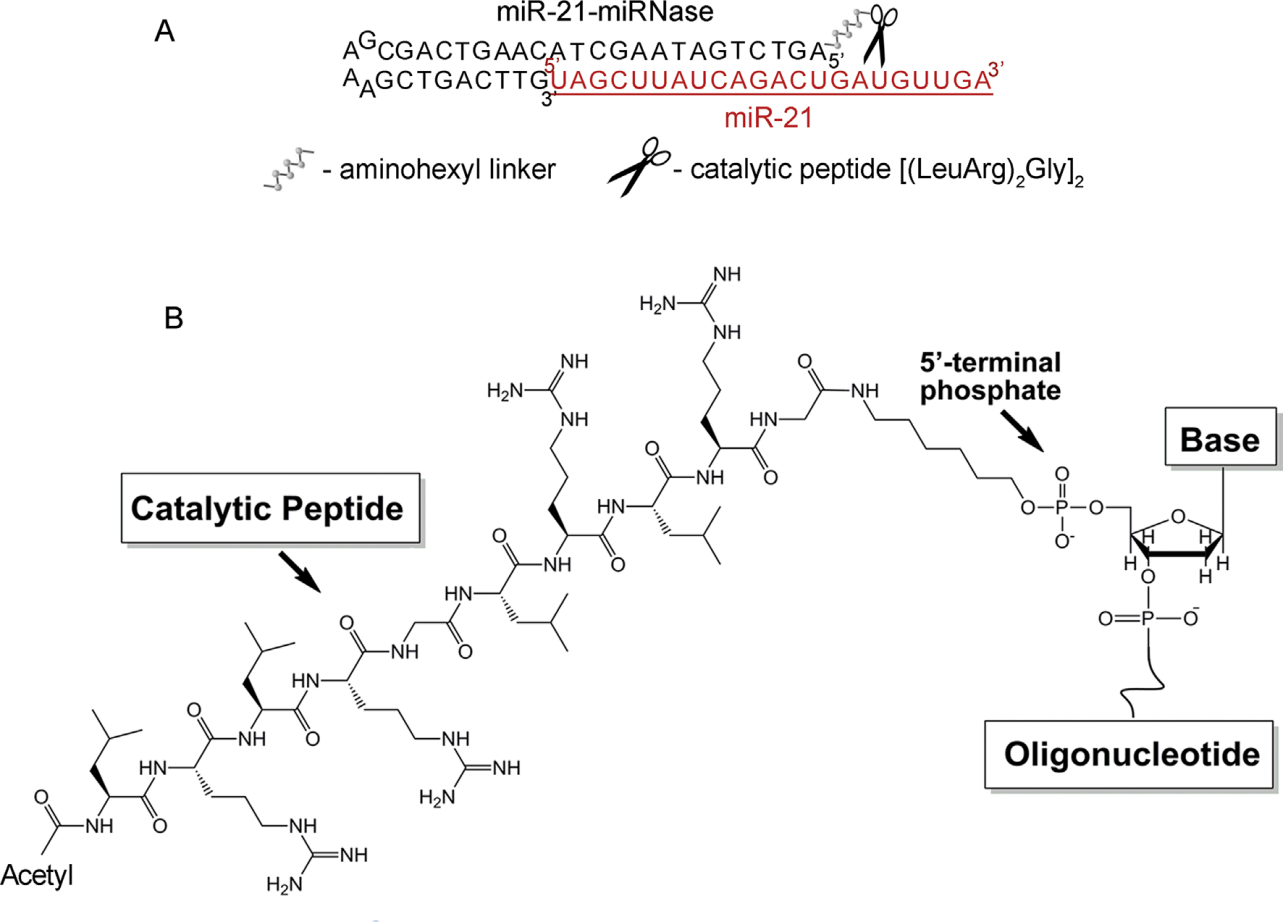
Structure of the miR-21-targeted peptide-oligonucleotide conjugate, miR-21-miRNase. **(A)** Sequence of oligonucleotide part of miRNase and schematic representation of a general complex of miR-21 and miRNase. **(B)** Chemical structure of miRNase. The peptide acetyl-[(LeuArg)_2_Gly]_2_ was covalently attached via its C-terminus to the aminohexyl linker located at the 5′-terminal phosphate of the antisense oligonucleotide.

Stability of antisense oligonucleotides (AOSs) against nucleases in serum is essential for *in vivo* application, which typically requires nuclease-resistant chemically modified nucleotides. We avoid this here by design of a nuclease-resistant structure with a hairpin at the 3′-end and the catalytic peptide at the 5′-end. The hairpin oligonucleotide (h-ODN) comprised a 14-mer recognition motif at the 5′-terminus, which is complementary to the miRNA target, and a hairpin structure with a four-member apical loop and 9 nts stem at its 3′-terminus. The catalytic peptide, acetyl-[(LeuArg)_2_Gly]_2_, was covalently attached *via* its C-terminus to the 5′-end of the oligonucleotide through an aminohexyl linker (**Figure 1**). The biological and therapeutic properties of miR-21-miRNase was compared with the properties of miR-21-targeted hairpin deoxyribooligonucleotide (h-ODN) and 2′O-methyl-modified oligonucleotide (2′-OMe) unconjugated with the peptide, with a linear 14-mer deoxyribooligonucleotide (ODN) and with the control conjugate (luc-POC), in which the sequence of the hairpin was the same but the complementary part was replaced by the fragment of the luciferase gene with no homology in the mammalian genome (**Supplementary Table 1**). Although the free oligonucleotide (ODN) was chemically unmodified and rapidly degraded in serum, the hairpin ODN resisted dilute serum for a few hours but survived for over 48 h (see **Supplementary Figure 1**), when conjugated to the catalytic peptide within the miRNase (**Supplementary Figure 1**).

### Induction of Apoptosis and Suppression of Invasiveness of Tumor Cells

**Figure 2:**
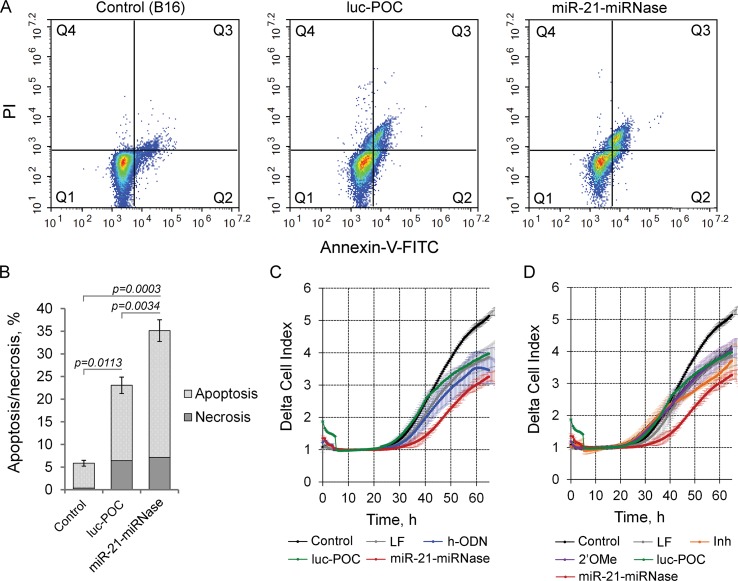
Biological effect of the miR-21-specific miRNase. **(A)** Apoptotic profile of B16 melanoma cells after transfection with miR-21-miRNase and control luc-POC. Cytofluorimetric analysis of cells after staining with Annexin V-FITC/PI 48 h after transfection with miRNase or luc-POC (1 µM) in complex with Lipofectamine2000. Q1—population of living cells; Q2—Annexin V-FITC+/PI-, early apoptosis; Q3—Annexin V-FITC+/PI+, late apoptosis; Q4—Annexin V-FITC-/PI+, necrosis. **(B)** Average percentage of apoptotic (early and late apoptosis) and necrotic B16 cell population. Data represent mean ± s.e. of four independent experiments.**(C, D)** Invasion activity of B16 cells after incubation with Lipofectamine (LF) alone or treated with either hairpin antisense oligonucleotide (h-ODN), hairpin 2′-OMe oligonucleotide (2′-OMe), commercial miR-21 inhibitor (Inh), miR-21-miRNase, and control conjugate (luc-POC) in the concentration of 1 µM. Migration of cells was recorded in real time using the xCELLigence cell analysis system. Control—B16 cells without treatment. Data represent mean ± s.d. of four independent experiments.

By modulating tumorigenesis through the regulation of multiplicity of critical genes suppressing malignant growth, evasion from apoptosis, invasion, and metastasis, miR-21 functions as an oncogene (Melnik, 2015). Knockdown of miR-21 by miR-21-miRNase resulted in specific elevation of the level of the direct mRNA target of miR-21, tumor-suppressor programmed cell death 4 (PDCD4) (Patutina et al., 2017a), and so may also turn down the malignant behavior of tumor cells through induction of apoptosis and inhibition of invasion and metastasis. We studied this in B16 melanoma cells, which are known for maintaining aggressive growth, active migration, and metastasis. As indicated by the Annexin V-FITC/PI assay, miR-21-miRNase increased necrosis of melanoma B16 cells to a limited extent (up to 7%) similar (up to 6.5%) to the sham peptide-oligonucleotide conjugate, luc-POC (for luc-POC sequence see **Supplementary Table 1**) with a sequence of a firefly luciferase gene with no complementarity to the mammalian genome. However, miR-21-miRNase caused a significant increase in the proportion of cells in the apoptotic state with a maximum at 48 h after transfection: the signs of apoptosis were observed in 28% of the cells (cf 15% control), which was six times higher than that in the intact B16 cells (**Figures 2A, B**, **Supplementary Figure 2**).

Cells transfected with h-ODN (blue curve), or its methylated derivative 2′-OMe (violet curve) or the sham luc-POC (green curve), migrated at almost the same rate as cells incubated with Lipofectamine (grey curve) (**Figures 2C, D**). However, miR-21-miRNase (red curve) strongly inhibited the invasion potential of B16 cells by about 70% relative to control and sham luc-POC (*p*=0.0008). (**Figures 2C, D**, time point 40 h). Knockdown of miR-21 by commercial Inh (orange curve) showed only a delayed inhibition of cell invasion of approximately 40% relative to control and only 20% relative to luc-POC.

### Persistence to Suppress Tumor Growth

**Figure 3:**
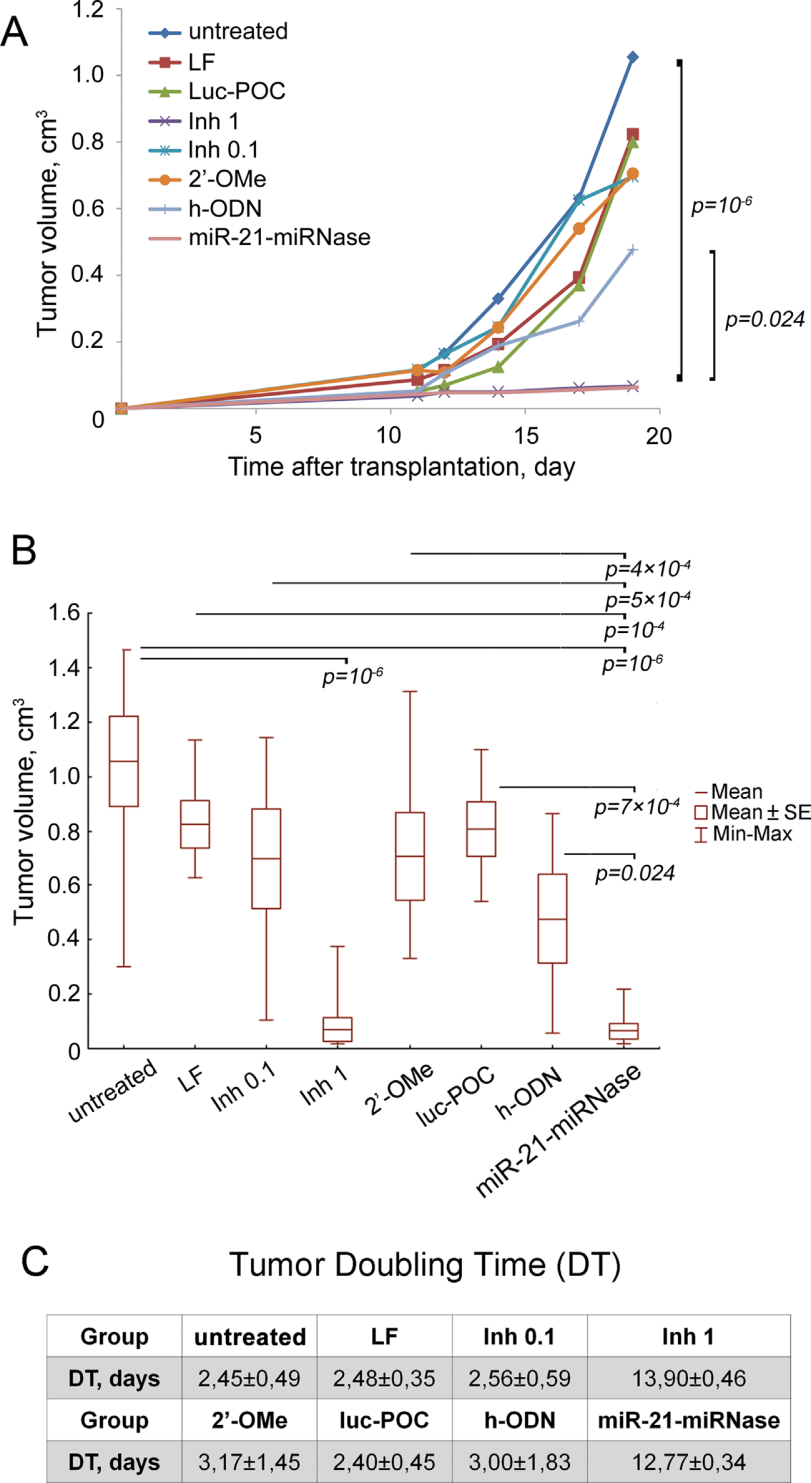
Antitumor effect of miR-21-specific miRNase. **(A)** Retardation of RLS_40_ growth *in vivo* after treatment of RLS_40_ cells with antisense oligonucleotides, control conjugate (luc-POC), or miR-21-miRNase. **(B)** Tumor volume on day 19 after RLS_40_ transplantation. Mice were injected with RLS_40_ cells either without any treatment (w/t) or treated with Lipofectamine (LF), commercial miR-21 inhibitor at concentration 0.1 µM (Inh 0.1, recommended) and 1 µM (Inh 1, in accordance to concentration of miRNase used in the study), control conjugate (luc-POC, 1 µM), hairpin 2′OMe oligonucleotide (2′-OMe, 1 µM), hairpin antisense oligonucleotide (h-ODN, 1 µM) or miR-21-specific miRNase (miR-21-miRNase, 1 µM). Data were statistically analyzed using two-way ANOVA with *post hoc* Tukey test. Data are presented as mean ± s.e. *p*-value indicates a statistically reliable difference. **(C)** Doubling time of RLS_40_ tumors after treatment with oligonucleotides and conjugates.

To evaluate the ability of miRNase to suppress growth of tumor cells and dissemination in the body, an experiment in mice was conducted. RLS_40_ lymphosarcoma cells were treated with the miRNase *in vitro* and then intramuscularly transplanted to the right lower limb of CBA mice to form solid tumors with dissemination in the body. miR-21-miRNase efficiency was assessed in a specially collected portion of the treated cells: a 2-fold decrease in miR-21 level by miR-21-miRNase was confirmed by PCR analysis (24 h after transfection), and 1.7- and 2.4-fold increase in protein levels of miR-21 target proteins—PTEN and PDCD4—was shown by Western blot hybridization, respectively, (72 h after transfection) (**Supplementary Figure 3**) which is consistent with previously obtained data (Patutina et al., 2017a). Despite no further treatment of the cells or animals transplanted, the effects of miR-21-miRNase persisted to retard subsequent tumor growth, with a mean tumor volume 16.7 times less than in the control group, showing a 94% tumor volume reduction upon treatment (**Figures 3A, B**), which was a statistically reliable difference (*p* value was 10^−6^ against control). Similar inhibition of tumor growth could only be seen when dosing of the positive controls with Inh was much elevated (1 µM, **Figures 3A, B**) to a 10- to 20-fold higher level than the manufacturer’s recommendations (0.05–0.1 μM). Whether the positive control was then specific or caused by a non-specific cytotoxic effect of Inh is unclear. The higher rate of tumor growth in the groups transplanted with RLS_40_ cells with no treatment (w/t), cells treated with Lipofectamine (LF) alone, Inh (0.1 μM), the methylated h-ODN 2′-OMe (1 μM), and luc-POC did not differ, and h-ODN retarded primary tumor growth at the limit of statistical significance (*p* ∼ 0.05) by only about 2-fold (**Figures 3A, B**).

Tumor doubling time (DT) was increased to 12.77 ± 0.34 days by miR-21-miRNase, >5-fold greater than of untreated control (w/t) of 2.45 ± 0.49 days (**Figure 3C**). The groups LF, 2′-OMe, luc-POC and h-ODN did not show a statistically significant difference against the w/t, and neither did the positive control of Inh, until a much (10×) elevated level was used to increase DT to a similar extent (13.90 ± 0.46 days). Relative liver weights remained similar to healthy animals for the miR-21-miRNase and excessive Inh treatments (see **Supplementary Table 2**), even though absence of metastases in the liver may not be expected to persist in tumors seeded from cells treated only before transplant.

### Synergistic Catalytic Recruitment of RNase H

**Figure 4:**
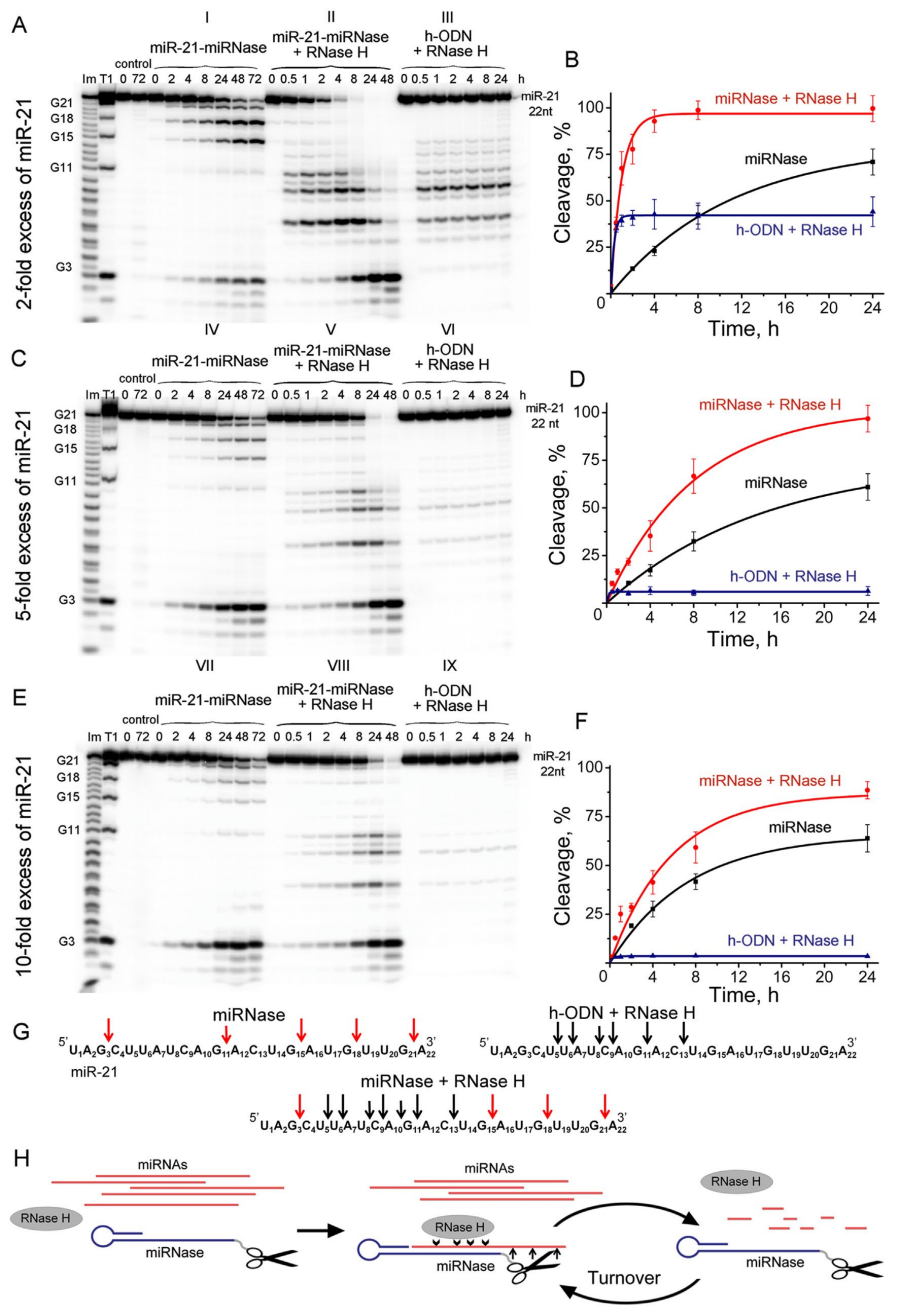
Cleavage of 5’-[^32^P]-labeled miR-21 by miR-21-miRNase and/or RNase H. Autoradiographs of 18% polyacrylamide/8 M urea gel, showing the patterns of cleavage of miR-21 at its 2- **(A)**, 5- **(C)**, and 10-fold **(E)** molar excess over miR-21-miRNase or h-ODN. Autoradiographs I, IV, and VII represent cleavage of miR-21 by miR-21-miRNase alone, whereas autoradiographs II, V, and VIII show cleavage of miR-21 by a mixture of miR-21-miRNase and RNase H. Autoradiographs III, VI, and IX demonstrate cleavage of the complex formed between miR-21 and h-ODN by RNase H alone (100 U/ml). Duplexes formed by 5′-[^32^P]-miR-21 (10, 25, and 50 µM) and h-ODN or miRNase (5 µM) were incubated at 37°C for 24–72 h. Lanes Im and T1: imidazole ladder and partial RNA digestion with RNase T1, respectively; “control”: RNA was incubated in the absence of oligonucleotide or conjugate and in the presence of RNase H (100 U/ml). Diagrams **(B)**, **(D)**, and **(F)** show time dependency of cleavage of miR-21 by miR-21-miRNase alone, by a synergetic action of miR-21-miRNase and RNase H, or by RNase H (100 U/ml) alone when miR-21 was in a complex with h-ODN at 2-, 5- and 10-fold molar excess of miR-21 over miR-21-miRNase or h-ODN, respectively. **(G)** Positions of miR-21 cleavage induced by miR-21-miRNase (red arrows), RNase H in the duplex with h-ODN (black arrows), and by combination of miR-21-miRNase and RNase H. **(H)** The hypothetical representation of miR-21 cleavage by a combination of miRNase and RNase H.

It is desirable that a true artificial nuclease exhibits a reaction catalytic turnover as a result of eventual release of the cleaved RNA fragments followed by attack of the next target miRNA molecules. Using the synthetic 5′-[P^32^]-labeled miR-21, we evaluated the ability of miRNase to cleave multiple copies of miR-21, when significant excess (up to 10-fold) of miRNA target was used over miR-21-miRNase. Since the recognition motif of miR-21-miRNase was represented by the “naked” (e.g., unmodified) oligodeoxyribonucleotide, it was anticipated that in cellular environment the duplex formed between the miRNase and miR-21 can potentially be recognized by RNase H, which is present in virtually all cells. Therefore, we aimed to evaluate the mutual effect of both RNases on each other (RNase H and miRNase) by comparing their individual activity against miR-21 with their joint action at various excess of miRNA target over miRNase. To achieve this, we ran nine series of the parallel cleavage assays (see **Figures 4A–G**). Series A(I), C(IV), and E(VII) (**Figure 4**) measured the efficiency of miRNA cleavage by miRNase alone at the 2-fold, 5-fold, and 10-fold excess of the target RNA with respect to miRNase, respectively. Series A(III), C(VI), and E(IX) evaluated the cleavage efficiency of the RNase H alone towards the miR-21 target present at the same concentrations, while it was hybridized into a heteroduplex with the corresponding oligonucleotide h-ODN lacking the catalytic peptide. Series A(II), C(V), and E(VIII) measured the combined action of miRNase and RNase H present together in the reaction.

We compared the cleavage patterns and kinetics of the miR-21-miRNase with the non-sequence selective RNase H, which is present in virtually all cells. miR-21-miRNase alone cleaved miR-21 at the G3C4, G15A16, G18U19, and G21A22 sites (**Figures 4A**-I, **C**-IV, and **E**-VII), whereas RNase H cuts the target predominantly at the U6A7, C9A10, and G11A12 sites of miR-21 (**Figures 4A**-III, **C**-VI, and **E**-IX). Neither of these cleavage patterns was affected by the concentration of miR-21. The respective activities of miR-21-miRNase and RNase H in combination (**Figures 4A**-II, **C**-V, and **E**-VIII) provided clear evidence that the hetero-complex formed by miRNA and miRNase is also a substrate for RNase H. Target miRNA was effectively cleaved by RNase H when heteroduplexed to the hairpin targeting structure, whether alone (h-ODN) or when conjugated to the peptide (miR-21-miRNase). Moreover, miRNA cleavage occurred at both G-X sites, which are specific for miRNase, and at the sites sensitive to RNase H.

At 2-fold molar excess of miR-21 with respect to h-ODN (**Figure 4A**), the kinetics of miRNA cleavage in a heteroduplex with h-ODN by RNase H alone reached a plateau by 30 min of incubation, but with a total extent of cleavage of only 45% (**Figures 4A**-III, **B**—blue curve). Moreover, with an increase in miRNA concentration to 5- and 10-fold excesses (**Figures 4C, E**), the extent of miRNA cleavage by RNase H fell to about 10% and 5%, respectively (**Figures 4C**-VI, **E**-IX; **D** and **F**—blue curves), which presumably reflected the lesser proportions of the miRNA/h-ODN heteroduplex within the overall amount of miRNA present. However, the extent of miRNA cleavage by the miRNase alone was higher and clearly indicative of catalytic turnover. Indeed, the level of cleavage of miR-21 by miRNase alone was similar irrespective of the 2-, 5-, or 10-fold excess of the target over miRNase and progressed towards 65–68% over 24 h (**Figures 4A**-I, **C**-IV, **E**-VII; **B**, **D** and **F**—black curves), and 90% over 72 h (**Supplementary Figure 4A**). Clearly, this miR-21-miRNase structural design was capable of cleaving many copies of the target miRNA, with a greater efficiency than that achieved by a classical antisense pathway *via* RNase H recruitment.

However, as RNase H is ubiquitous in target cells, we also considered whether the simultaneous presence of RNase H affected degradation of miR-21 by this miR-21-miRNase. We discovered that the presence of RNase H led to a considerable increase in the rate and extent of miRNA cleavage, as compared to the individual action of each enzyme alone under identical conditions (**Figure 4**, compare II with I and III, V with IV and VI, and VIII with VII and IX). At a 2-fold excess of miR-21 over miR-21-miRNase, complete (∼100%) RNA degradation was observed after 8 h of incubation (**Figures 4A**-II, **B**—red curve, **Supplementary Figure 4B**). With a 5-fold excess, such degradation took 24 h (**Figures 4C**-V, **D**—red curve, **Supplementary Figure 4B**), and with a 10-fold excess, RNA degradation was complete within 48 h (**Figures 4E**-VIII, **F**—red curve, **Supplementary Figure 4B**).

By interpolating the half-life (τ/2) of miR-21 degradation, we estimated the catalytic turnover in the absence and presence of RNase H. RNase H elevated cleavage of miR-21 by the miR-21-miRNase by a factor of 14.9 times in the case of 2-fold excess of miR-21, whereas this was less pronounced at 3.0 and 2.4 times for greater excesses of miR-21 at 5-fold and 10-fold, respectively (**Table 1**). In the absence of RNase H, the catalytic turnover of miR-21-miRNase alone was estimated to be around 100 cleaved RNA molecules per molecule of miRNase in 1 h in the cases of 2- and 5-fold excess of miR-21, but reached 382 cleaved RNA molecules per hour at 10-fold miR-21 excess (**Table 1**). The presence of RNase H enhanced the catalytic turnover by factors of 15.6, 7.0, and 4.6 times for 2-fold, 5-fold, and 10-fold excess of miR-21, respectively, with the potential to reach up to 1,800 cleaved miR-21 molecules by one miR-21-miRNase molecule per hour. A possible explanation of such a synergistic effect is that the combined action of the miRNase and RNase H led to formation of very short cleavage fragments of three nucleotides and shorter (**Figures 4A**-II, **4C**-V, **4E**-III) that may rapidly dissociate from the hybridized complex and release the sequence-specific miR-21-miRNase, when it would become available for another cleavage event (see **Figure 4H**). Indeed, the almost complete cleavage of miRNA at 5-fold excess formed short 2- and 3-nucleotide fragments (**Supplementary Figure 5**).

**Table 1:** The half-life time of miR-21 and reaction turnover in the presence of miR-21-miRNase and RNase H.

[miRNase], µM	[miR-21], µM	RNA excess	τ/2^1)^, h		Rt^2)^	
			RNase H		RNase H	
			–	+	–	+
5	10	2-fold	10.4 ± 0.5	0.7 ± 0.2	104 ± 10	1620±105
	25	5-fold	16.2 ± 1.5	5.4 ± 0.4	106 ± 11	740 ± 90
	50	10-fold	11.04 ± 0.8	4.7 ± 0.4	382 ± 28	1760±132

^1)^τ/2—a half-life time of miR-21 in the presence of catalysts, the time during which 50% of miRNA molecules is cleaved.

^2)^Rt, reaction turnover—the number of miRNA molecules cleaved by one molecule of miRNase per 1 h. Rt was calculated according to equation Rt = [miR-21] × (number of bonds) × (cleavage, %)/[miRNase].

## Discussion

Short functional non-coding microRNAs are implicated in many types of cancer (Frixa et al., 2015; O’Bryan et al., 2017; Zhou et al., 2017). Amongst many miRNAs identified as important inducers of oncogenesis, miR-21 represents an extreme oncomiR, which is strongly involved in tumor onset and progression for many types of malignancies (Huang et al., 2013; Lianidou et al., 2016; Yu et al., 2018). Since miR-21 is abnormally overexpressed in major types of tumors, this oncomiR is the most considered target for anti-miRNA inhibitory studies. However, depending on the origin of tissue and degree of pathological state, the concentration of miRNAs in the cell can vary from several copies to 2–4 μM concentrations, which can be too great to knock down effectively by antisense approaches alone. The most widely used anti-miRNA oligonucleotides, including commercial inhibitors fabricated on the basis of nucleic acids, are usually the perfect complements to miRNA targets, which contain various chemical modifications at the sugar-phosphate backbone to enhance their nuclease resistance and improve binding affinity. However, the chemical substitutions used for generation of such inhibitors are often not compatible with the activity of intracellular RNase H, and their inhibitory effect relies on 1:1 stoichiometry of binding between the oligonucleotide-based inhibitor and miRNA target. Although the use of such oligonucleotide-based inhibitors shows detectable level of miRNA silencing, the absence of the catalytic amplification represents their disadvantage as compared with our biocatalytic agents, capable to degrade irreversibly multiple copies of pathogenic miRNA molecules, which will be particularly crucial to effectively suppress malignant growth. On the basis of our recent discovery of peptide-oligonucleotide conjugates (Patutina et al., 2017a; Staroseletz et al., 2017; Patutina et al., 2018) with desired biocatalytic properties against disease-relevant RNAs, we have designed miRNA-specific aRNase targeted to miR-21. Efficient knockdown of miR-21 by the miR-21-miRNase triggered a broad spectrum of biological responses, leading to the inhibition of pro-survival behavior of tumor cells, including inhibition of tumor cell proliferation by 50% (Patutina et al., 2017a), the initiation of apoptosis in 28% of the tumor cell population, and suppression of cell invasiveness by 60–70%. Furthermore, upon transplantation of the treated tumor cells into mice, the oncosuppressive effect of miR-21-miRNase persisted. Even a single treatment of tumor cells with miR-21-miRNase prior to transplantation led to a 50% reduction in miR-21 level and a drastic (94%) reduction in primary tumor growth in mice. Tumor doubling time increased by more than 5-fold (from 2.45 to 12.77 days) for tumor cells treated with miR-21-miRNase prior to transplantation, as compared to control groups without treatment or treated with a sham (luc-POC) or the hairpin oligonucleotide (h-ODN). The biological effects induced by this miRNase are comparable or superior to those of available miR-21 oligonucleotide inhibitors (Dong et al., 2012; Wagenaar et al., 2015; Wu et al., 2016; Wang et al., 2018; Zhou et al., 2018).

The design developed for this miRNase: i) enhanced the hybridization efficiency towards the miRNA target, probably due to the additional stacking interactions provided by the hairpin structure (Sunami et al., 2004; Patutina et al., 2017b); ii) bound only mature forms of miRNA; and iii) protected the oligonucleotide from nuclease degradation. It is well known that high nuclease susceptibility of natural oligodeoxyribonucleotides leads to short half-life and reduced antisense functionality *in vivo*, typically requiring the use of chemically modified oligonucleotides to avoid this rapid degradation. However, the rational design of nuclease resistance into these therapeutic miRNases was most likely provided by the hairpin targeting structure and the catalytic peptide location. The h-ODN with a hairpin at the 3′-end remained intact for a few hours but, with the catalytic peptide conjugated at the 5′-end of the hairpin oligonucleotide, persisted for more than 24 h. This nuclease resistance of the miR-21-miRNase undoubtedly contributed to the longer and stronger biological effect observed *in vitro* and *in vivo*. The nuclease resistance of this miRNase is not inferior to the stability of potent phosphorothioate DNA/LNA mixmer, which begins to degrade by 24 h of similar incubation (Lennox and Behlke, 2010). The obtained results correlate with the data reported earlier, where it was shown that 3′-end structures can improve resistance to snake venom phosphodiesterase, DNA polymerase I, and FBS and significantly increase functional potency for target RNA knockdown (Tang et al., 1993; Hosono et al., 1995). The presence of flanking duplexes also improves the ability of anti-miRNA oligonucleotides to invade RISC and serve functional enhancement (Vermeulen et al., 2007; Lennox and Behlke, 2011).

Clear evidence of the catalytic nature of miRNA cleavage by this miRNase was found when the miR-21 was present at 2-, 5-. or 10-fold excess over the miRNase. In contrast, RNase H alone did not exhibit any catalytic turnover, as the extent of miR-21 cleavage by this ribonuclease simply correlated with the proportion of the miR-21/h-POC hetero-complex within the overall amount of miRNA present, which is the expected result for a conventional antisense-mediated pathway involving RNase H recruitment. Interestingly, neither introduction of the hairpin nor the presence of the peptide within the structure of the miR-21-miRNase affected the ability of RNase H to recognize and cleave miR-21 hybridized with the oligonucleotide moiety of the miR-21-miRNase.

Particularly exciting though was the discovery of the synergistic effect from the joint action of this miRNase and RNase H towards miR-21. In isolation, both RNases cleaved totally different regions of miR-21, which can be easily identified from the PAAG analysis (**Figure 4**). However, when miR-21 formed a complementary duplex with h-ODN oligonucleotide, RNase H recognized this hetero-complex and cleaved miRNA at the “seed region,” located close to the 5′-end (bases 2–8), which is known to be a “canonical” determinant in miRNA function (Lai, 2002). In contrast, miR-21-miRNase cleaved the 3′-region of the miR-21 molecule (bases 13–18), which represents a “3′-compensatory” or “beneficial 3′-paring” site in the miRNA-mRNA target recognition and plays a crucial role for miRNA specificity and functioning (Brennecke et al., 2005; Grimson et al., 2007; Wang et al., 2009; Robertson et al., 2010). However, when both ribonucleases were present (which appears likely *in vivo* in the intracellular environment), we observed a significant increase of the rate of miRNA cleavage. Earlier, using miRNA-targeted conjugates of a different structure, we have shown that the presence of RNase H can significantly enhance its activity and increase the rate of target RNA cleavage, without any positive effect on RNase H activity (Patutina et al., 2018). In this study, we discovered a different mechanism of synergy when both RNases showed mutual enhancement of their cleavage activity in the presence of each other, which was considerably higher than any simple additive effect expected from independent enzymatic action. The observed synergistic effect from this combined attack on miRNA molecules could be attributed to multiple cuts within miR-21 induced by these ribonucleases, thus leading to a formation of short cleavage products. This may trigger a rapid collapse of the hybridized complex and a release of both miRNase and RNase H, when each could then initiate a new attack on another miR-21 molecule (**Figure 4H**). This mechanism may well explain the high level of inactivation of oncogenic miRNA and the persistence of diverse suppression of malignant behavior of tumor cells upon treatment with this miR-21-miRNase.

We conclude that high therapeutic efficacy of peptide-oligonucleotide conjugates is likely to arise from the following: 1) high resistance to nuclease degradation from structural design, 2) efficient cleavage of miRNA-target molecules in a multiple turnover or true catalytic manner, and 3) by synergistically harnessing intracellular RNases such as RNase H. Over the past few decades, many different artificial ribonucleases have been reported, with some cleaving RNA *in vitro* with high efficiency and selectivity. However, to the best of our knowledge, we report here the first example of metal-independent, RNA-specific peptide-oligonucleotide conjugates operating under physiologically relevant conditions within tumor cells, which maintained miRNA knockdown in tumors subsequently formed from cells transplanted into mice, without further treatment. These findings elevate these sequence-specific aRNases to worthy rivals to antisense and siRNA technologies for the development of new RNA targeting therapeutics.

The key discoveries of this research provide a solid experimental ground for facilitating a paradigm shift in the development of new therapeutic interventions to ultimately offer effective, safe and cost-efficient treatment of unresectable or metastatic tumors, which currently relies on the cytotoxic effect of chemotherapies or radiotherapy and inevitably leads to a severe toxicity in humans. A fundamental change in treatment from small-molecule anti-cancer drugs, which often suffer from poor target selectivity, to highly selective RNA-targeting therapies provides a possibility to overcome such undesirable side effects arising from cytotoxic drug cocktails of combination regimens currently used in oncology. Our miRNA-selective chemical ribonucleases offer unprecedented opportunity to selectively knock down many copies of oncogenic miRNA in cells by irreversible cleavage in a truly catalytic manner with multiple turnovers, which is reinforced even further by recruitment of intracellular RNase H. This will ultimately boost potency, reduce dosage, and decrease cost of treatment. In the future, miRNases can be used both as a monotherapy and as an adjuvant therapy in combination with surgical treatment, radiotherapy, and chemotherapy. In the latter case, miRNases may allow to significantly decrease the dosage of cytostatics thereby reducing toxicity. Application of miRNases is a promising and rapidly evolving area of antisense technology, and its suitable combination with chemotherapeutics may represent highly efficient approaches to treating oncopathologies and other miRNA-associated diseases in humans.

## Data Availability

All datasets generated for this study are included in the manuscript/supplementary files.

## Ethics Statement

This study was carried out in accordance with the international recommendations of the European Convention for the Protection of vertebrate animals used for experimental studies (1997), as well as the rules of laboratory practice in the performance of pre-clinical studies in the Russian State Standards (R 51000.3–96 and 51000.4–96), the Committee on the Ethics of Animal Experiments with the Institute of Cytology and Genetics of the Siberian Branch of the Russian Academy of Sciences. The protocol was approved by the Committee on the Ethics of Animal Experiments with the Institute of Cytology and Genetics of the Siberian Branch of the Russian Academy of Sciences.

## Author Contributions

MZ, EB, and OP conceptualized and designed the study. VV supervised the research. OP, SM, NM, AS, and EB performed the experiments and analyzed the data. OP, EB, DC, and MZ interpreted the data, wrote, edited, and revised the manuscript. All authors approved the final version of the manuscript.

## Funding

This work was funded by Russian Science Foundation (Grants No. 14-44-00068 and No. 19-14-00250) and by Russian State funded budget project of ICBFM SB RAS # АААА-А17-117020210024-8, BBSRC (Grant No. BB/K012622/1), and EPSRC (Grant No. EP/E003400/1).

## Conflict of Interest Statement

The authors declare that the research was conducted in the absence of any commercial or financial relationships that could be construed as a potential conflict of interest.
