# Supplementary material for: Catalytic Knockdown of miR-21 by Artificial Ribonuclease: Biological Performance in Tumor Model
Source: Front Pharmacol. 2019 Aug 8;10:879. doi: 10.3389/fphar.2019.00879 (PMC6698794; doi:10.3389/fphar.2019.00879)
Supplement: Supplementary file 1 [file Presentation_1.pdf]

Supplementary Material

Supplementary Table 1. Oligonucleotides and peptide-oligonucleotide conjugates (POC) used in the study: sequences and nomenclature

Name, length	Oligonucleotide sequence (5' → 3')
**ODN**	TCAGTCTGATAAGC
**h-ODN**	AGTCTGATAAGCTACAAGTCAGCGAAAGCTGACTTG
**2'-OMe**	$A_mG_mT_mC_mT_mG_mA_mT_mA_mA_mG_mC_mT_mA_mC_mA_mA_mG_mT_mC_mA_mG_mC_mG_m$ $A_mA_mA_mG_mC_mT_mG_mA_mC_mT_mT_mG_m$
**miR-21-miRNase**	acetyl-[(LeuArg)$_2$Gly]$_2$-aminohexyl-AGTCTGATAAGCTACAAGTCAGCGAAAGCTGACTTG
**luc-POC**	acetyl-[(LeuArg)$_2$Gly]$_2$-aminohexyl-CGATAAATAACGCGCAAGTCAGCGAAAGCTGACTTG
**Inh**	hsa-miR-21 5p Inhibitor, Ambion, USA

Regular font – sequence complementary to miR-21, except for luc ODN; underlined font – a hairpin; N_m – 2'-OMe nucleotide.

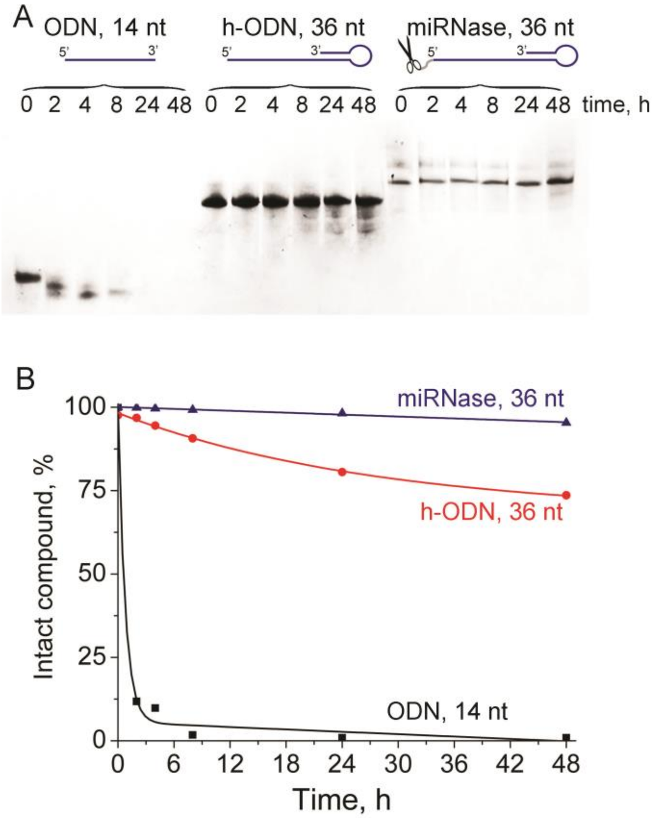

Supplementary Figure 1. Stability of the miR-21-miRNase in serum. **(A)** Kinetics of degradation of the 14-mer oligodeoxyribonucleotide (ODN, 14 nt), hairpin oligodeoxyribonucleotide (h-ODN, 36 nt), and miRNase (miRNase, 36 nt), consisting of a hairpin oligonucleotide and a catalytic peptide attached at its 5'-terminus in 10% FBS at 37°C. Image of the 12% PAAG/8M urea, stained with Stains-All. **(B)** Secondary plot of the data shown in **(A)**.

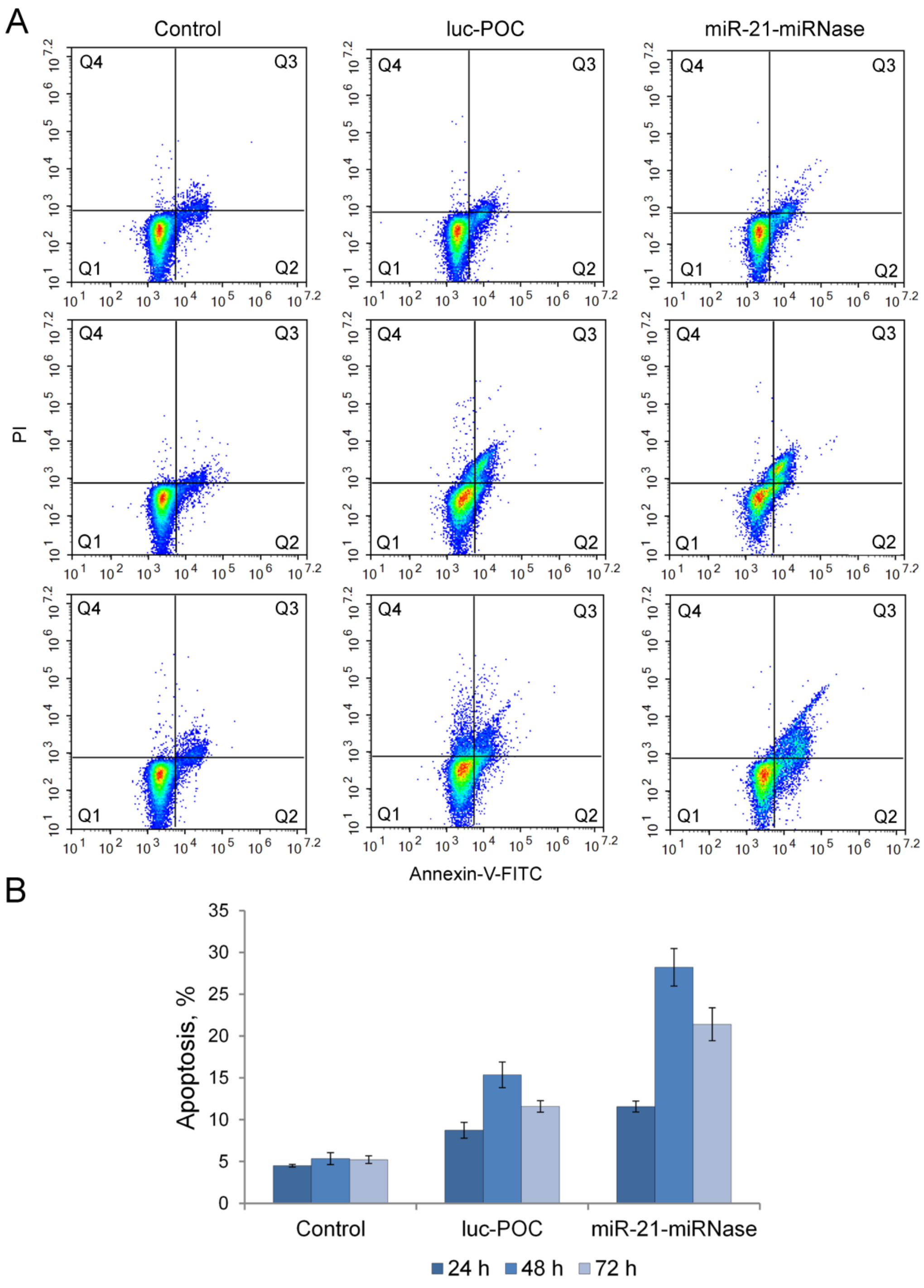

Supplementary Figure 2. Apoptotic effect of miR-21-miRNase. **(A)** Apoptotic profile of B16 melanoma cells after transfection with miR-21-miRNase and control luc-POC. Cytofluorimetric analysis of cells after staining with Annexin V-FITC/PI 24, 48 and 72 h after transfection with miRNase or luc-POC (1 μM) in complex with Lipofectamine^{TM} 2000. Q1 – population of living cells; Q2 – Annexin V-FITC+/PI-, early apoptosis; Q3 – Annexin V-FITC+/PI+, late apoptosis; Q4 – Annexin V-FITC-/PI+, necrosis. **(B)** Average percentage of apoptotic (early and late apoptosis) and necrotic B16 cell population. Data represent mean ± s.e. of four independent experiments.

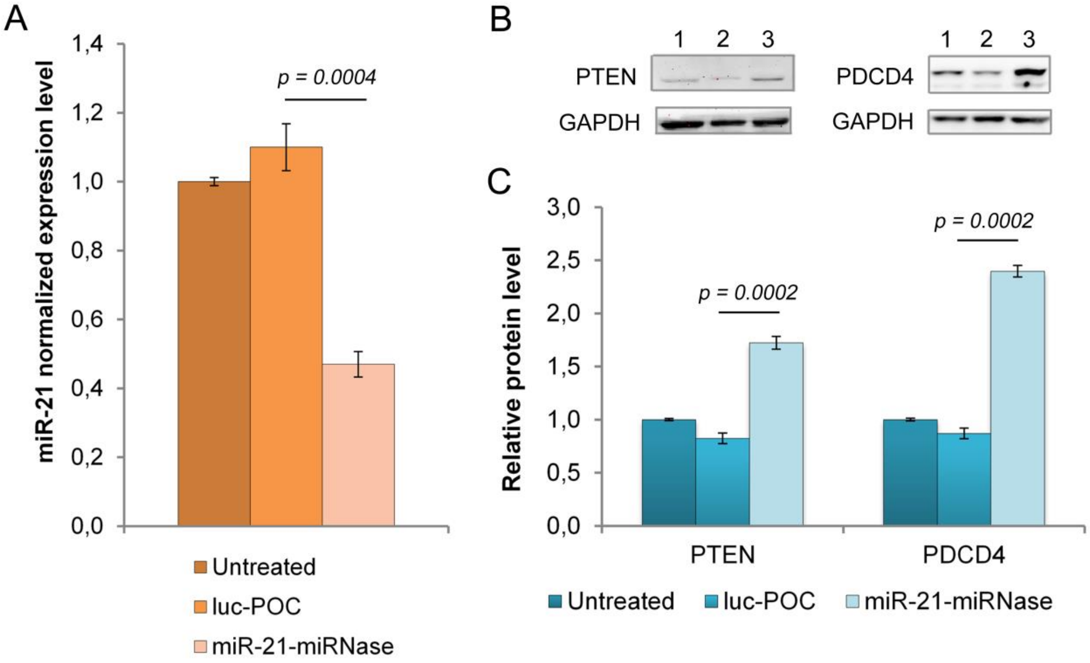

Supplementary Figure 3. Expression level of miR-21 and PTEN and PDCD4 protein level in RLS_{40} cells after transfection with 1 μM of luc-POC or miR-21-miRNase using Lipofectamine^{TM} 2000. **(A)** Expression level of miR-21 24 h after transfection. The expression of miRNAs was normalized to U6. **(B)** Representative pictures of western blot analysis of PTEN and PDCD4 proteins 72 h after transfection. GAPDH served as an internal control. 1 – untreated RLS_{40} cells; 2 – RLS_{40} cells transfected with luc-POC; 3 – RLS_{40} cells transfected with miR-21-miRNase. **(C)** The bar graph shows the semi-quantitative analysis of the western blot results for PTEN and PDCD4. Data are presented as mean ± SE.

Supplementary Table 2. Hepatic indexes of mice implanted with RLS_{40} treated with antisense oligonucleotides, control luc-POC and miR-21-miRNase

Type of treatment	Hepatic index, %*	*p*-value (*vs* control)
w/t	7.76±1.11	
LF	6.02±0.55	s.i.
luc-POC	5.56±0.11	*0.009*
Inh 1	5.17±0.18	*0.001*
Inh 0.1	5.67±0.16	*0.031*
2'-OMe	5.26±0.18	*0.005*
h-ODN	5.23±0.17	*0.004*
miR-21-miRNase	5.1±0.16	*0.001*

* – Mean ± SE; s.i. – statistically insignificant

Hepatic index was estimated as (liver weight/mouse weight) × 100% and as 5.0% for healthy CBA mice.

The MDR status of RLS_{40} may add to the burden on the liver, when this type of tumor metastasizes into the liver (Sen'kova et al., 2012). *Ex vivo* treatment of RLS_{40} cells by oligonucleotides and miRNase avoids the toxic effect of antisense oligonucleotides, their conjugates and transfection reagents directly on the tumor-bearing animal. This may allow consideration of whether the decrease of migration activity of tumor cells translated into reduced metastatic burden of RLS_{40} on the liver. Treatment of the cells seeding the tumor may not reduce migration and metastases in the liver in the subsequent generations of cells forming tumors (albeit slower growing and smaller). However, *ex vivo* treatment with the miR-21-miRNase or Inh at elevated levels did allow the hepatic index to remain similar to healthy animals, but the control oligonucleotides (OMe, h-ODN) and peptide conjugate (luc-POC) also tended to retain a lower hepatic index.

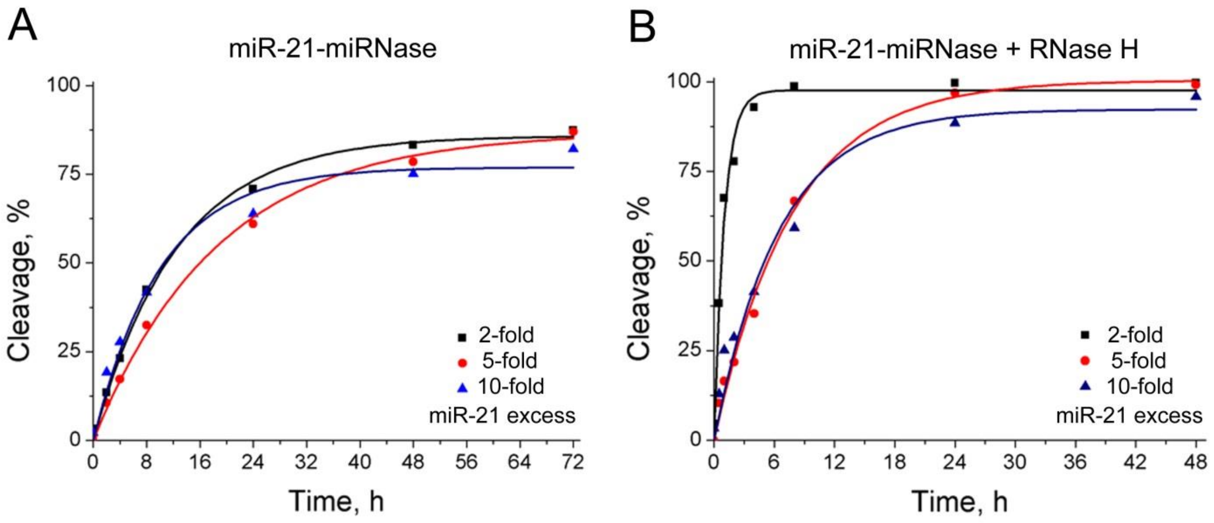

Supplementary Figure 4. Cleavage of excess of 5'-[P^{32}]-labeled miR-21 by miR-21-miRNase alone and in combination with RNase H. Kinetics of cleavage of 2-, 5-, and 10-fold excess of miR-21 by miR-21-miRNase **(A)** and by combination of miR-21-miRNase and RNase H **(B)**. miR-21 (10, 25 and 50 μM) and miRNase (5 μM) or miRNase (5 μM) and RNase H (100 U/ml) were incubated at 37°C for 48 – 72 h.

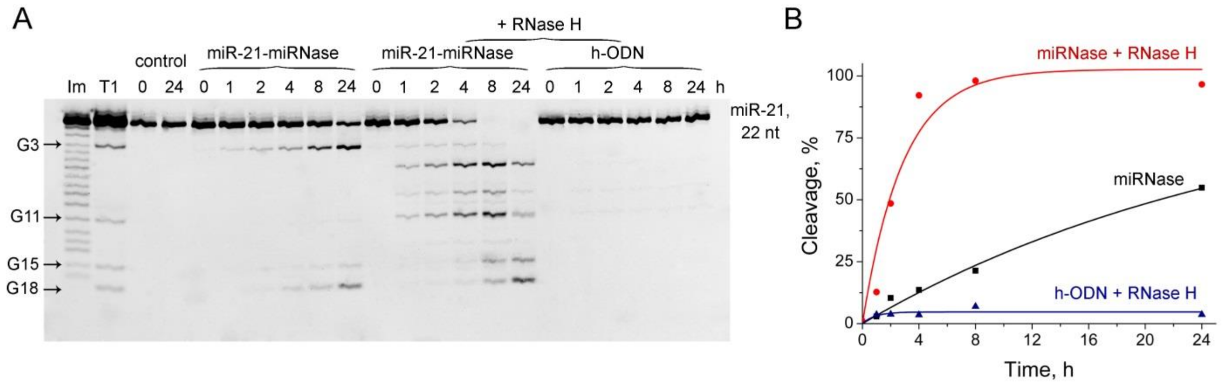

Supplementary Figure 5. Cleavage of excess of 3'-FITC-labeled miR-21 by miR-21-miRNase and RNase H. **(A)** Patterns of cleavage of 5-fold excess of miR-21 in complex with miR-21-miRNase alone, in complex with miR-21-miRNase by RNase H, or in complex with h-ODN by RNase H. Autoradiograph of 18% polyacrylamide/8 M urea gel. Duplexes of 3'-FITC-miR-21 (25 μM) and h-ODN or miRNase (5 μM) were incubated at 37°C for 24 h, RNase H was added at the concentration of 100 U/ml. Lanes Im and T1, imidazole ladder and partial RNA digestion with RNase T1, respectively; control – RNA was incubated in the absence of oligonucleotide or conjugate and in the presence of RNase H. **(B)** Kinetic dependency of cleavage 5-fold excess of miR-21 in complex with miR-21-miRNase alone, in complex with miR-21-miRNase by RNase H, or in complex with h-ODN by RNase H.

References

Sen'kova, A. V., Mironova, N. L., Patutina, O. A., Ageeva, T. A., and Zenkova, M. A. (2012). The Toxic Effects of Polychemotherapy onto the Liver Are Accelerated by the Upregulated MDR of Lymphosarcoma. *ISRN Oncol.* 2012, 1–15. doi:10.5402/2012/721612.
